# PSYCHOMETRIC PROPERTIES OF THE NEUROPSYCHIATRIC INVENTORY QUESTIONNAIRE FOR THAI OLDER ADULTS WITH DEMENTIA

**DOI:** 10.1093/geroni/igac059.1510

**Published:** 2022-12-20

**Authors:** Kaipeng Wang, Ladson Hinton, Sue Levkoff, Siranee Sihapark, Komatra Chuengsatiansup, Hongtu Chen

**Affiliations:** University of Denver, Denver, Colorado, United States; University of California Davis, Sacramento, California, United States; University of South Carolina, Columbia, South Carolina, United States; Boromarajonani College of Nursing, Khon Kaen, Khon Kaen, Thailand; Society and Health Foundation & Sirindhorn Anthropology Center, Nonthaburi, Nonthaburi, Thailand; Harvard University, Boston, Massachusetts, United States

## Abstract

Psychiatric and behavioral symptoms are highly prevalent among persons living with dementia. Clinical evaluation of those symptoms is critical for assessment of dementia progression and development of effective treatment. The Neuropsychiatric Inventory Questionnaire (NPI-Q) is a brief informant-based instrument to assess the severity of 12 types of neuropsychiatric symptoms among persons with dementia and associated caregiver distress. Although Thailand is experiencing a rapid increase in the prevalence with dementia, there is no validated instrument to assess neuropsychiatric symptoms in Thailand. This study aims to examine the psychometric properties of the NPI-Q instrument among older adults with dementia in Thailand. Data were collected from 353 participants aged 60 and over with probable dementia, at least one neuropsychiatric symptom, and an adult family caregiver willing to participate in the study. We examined the internal consistency, factorial validity, and criterion validity of the NPI-Q instrument. NPI-Q has acceptable internal consistency (Cronbach’s α=0.77). Principal component analysis yielded a five-factor solution, which accounted for 63% of the variance. The five factors included (1) agitation, anxiety, and irritability, (2) delusions, hallucinations, apathy, and disinhibition, (3) euphoria, motor disturbance, and night disturbance, (4) depression, and (5) abnormal appetite. We found that higher NPI-Q severity score was significantly associated with higher geriatric depression, associated caregiver distress, caregiver burden, and caregiver depression and lower quality of life among older adults and their caregivers, which confirmed the criterion validity of the instrument. Findings provide support for use of the NPI-Q among older adults with dementia in Thailand.

